# Sudden Death of Mental Health Service Users During a Pandemic; a follow up study of consecutive unexpected deaths during the current pandemic compared to a previous consecutive cohort of persons who took their own lives

**DOI:** 10.1192/j.eurpsy.2022.2171

**Published:** 2022-09-01

**Authors:** C. Haley, R. Mcdaid

**Affiliations:** Donegal Mental Health Services, Letterkenny University Hospital, Dept Of Psychiatry, XDC, Ireland

**Keywords:** covid, Suicide, service user

## Abstract

**Introduction:**

The Covid pandemic has mental health consequences. This study examined service user suicides for thirteen months during the pandemic and “lock down” restrictions in Ireland .It compares variables from this group with a previous 2016 study examining service user suicides conducted in Ireland. Despite a previous trend of improving suicide there was an increase in people who were involved with mental health services completing suicide during the pandemic.

**Objectives:**

To hypothesize that social disequilibrium caused by the Covid pandemic and its control measures may remove the benefit of protective factors in suicide.

**Methods:**

The 2016 study used the Suicide Support and Information System- Psychological Autopsy Model as its methodology.The Rosenberg criteria were used to make a determination of suicide. This 2021 study used an anonymous clinical record review to repeat some key variables identified in the SSIS-PAM work. Statiscal comparisons were made.

**Results:**

The pandemic group had a different pattern of suicide with low levels of significance between four variables. The majority were female, in a relationship, had jobs, no history of self-harm, no family history of mental illness, less addiction problems and in-patient care. Like the 2016 group they did have diagnoses of mental illness, were in regular contact with services and were prescribed medication. Their suicides were predominantly hanging and drowning.

**Conclusions:**

During the pandemic suicide increased. Protective factors such as relationships and employment were increased in pandemic suicides who also had less vulnerability factors such as addiction problems and self-harm. “Real time” suicide data collection such as a suicide observatory model might identify more significant trends.

**Disclosure:**

No significant relationships.

